# The complete chloroplast genome and phylogenetic analysis of *Cardamine circaeoides* Hook. f. et Thoms., 1861 (Brassicaceae)

**DOI:** 10.1080/23802359.2022.2141081

**Published:** 2022-11-15

**Authors:** Ru Wang, Zhijun Deng, Yongjian Luo

**Affiliations:** a Hubei Key Laboratory of Biologic Resources Protection and Utilization, Hubei Minzu University, Enshi, P. R. China; b Research Center for Germplasm Engineering of Characteristic Plant Resources in Enshi Prefecture, Hubei Minzu University, Enshi, P. R. China; c Center for Crop Germplasm Resources, Institute of Crop Sciences, Chinese Academy of Agricultural Sciences, Beijing, P. R. China

**Keywords:** *Cardamine circaeoides*, chloroplast genome, Brassicaceae, sequence alignment

## Abstract

A species of *Cardamine circaeoides* Hook. f. et Thoms., 1861, which belongs to the Cardamine family (Brassicaceae), is an endemic species of the Wuling Mountains in Hunan and Hubei Provinces of China. Since there are many morphologically related species of *C. circaeoides*, the chloroplast (cp) genome characteristics of *C. circaeoides* were analyzed in order to explore the phylogenetic relationship between it and the closely related species. A cp genome totaling 154,838 base pairs exhibited a typical quadripartite structure with a pair of IRs (inverted repeats; 26,493 base pairs) separated by a small single-copy region of 17,938 base pairs and a large single-copy region of 83,914 base pairs. A total of 130 genes were found in the cp genome, including 85 protein-coding genes, 37 tRNA genes, and 8 rRNA genes. There were 36.21%, 33.96%, 29.09%, and 42.35% GC content in the entire cp genome, LSC region, SSC region, and IR region, respectively. According to phylogenetic analysis, *C. circaeoides* is evolutionarily closer to *Cardamine hupingshanensis*.

*Cardamine circaeoides* belongs to the Brassicaceae family. The species is primarily found in the high mountains, valleys, and streams of the Wuling mountains of Hunan and Hubei Provinces, China (Wu et al. 2020). Besides being rich in protein, soluble sugar, amino acids, cellulose, and other nutrients, it also provides a super enrichment of selenium (Tian and Cheng 2003). In the case of selenium supplement cultivation, organic selenium concentrations can reach 3553 mg/kgDW (Both et al. 2018) and its tender stems and leaves can be eaten. In the field survey, local residents often refer to it as ‘wild rape’, ‘water rape’, or ‘rock plate vegetable’, which is a highly nutritious wild vegetable. As technology continues to develop rapidly, scientists began to pay attention to the application of modern molecular techniques in order to characterize the genetic diversity of shepherd’s purses. It has been demonstrated in several research studies that chloroplast (cp) genome sequences play an important role in plant phylogeny and genetic analysis (Wang et al. 2016; Ma et al. 2021; Yu et al. 2021). *Cardamine circaeoides*’s cp genome has been reported for the first time, and its structural characteristics have been analyzed. This study provides important genetic and phylogenetic information for understanding this species’ genetic relationships.

This study examined the fresh leaves of *C. circaeoides* collected from Enshi City, Hubei Province, China (30.301 N, 109.487E, Altitude:1236 m). A specimen was deposited at the Guangdong Agro-biological Gene Research Center (http://multi-omics.agrogene.ac.cn/, contact person:Yongjian Luo, and email: 851022933@qq.com) under the voucher number 20210303005. An extraction of genomic DNA from dried silica leaves was performed using Qiagen’s DNeasy Plant Mini Kit. The 1% agarose gel and Thermo Scientific Nanodrop spectrophotometer were used to confirm the quality and quantity of the samples (Figure 1). A high-throughput sequencing platform (HiSeq 4000) was used to sequence the complete genomes of *C. circaeoides*. The paired-end sequence reads were trimmed to remove adaptors and low-quality sequences via Trimmomatic 0.39. Clean reads were assembled using GetOrganelle v1.5 (Jin et al. 2020). Chloroplast genome annotation was performed using CPGAVAS2 (http://47.96.249.172:16019/analyzer/home) (Shi et al. 2019) and GeSeq (https://chlorobox.mpimp-golm.mpg.de/geseq.html) (Tillich et al. 2017). An accession number (OL634846) has been assigned to the annotated cp genomic sequence in GenBank.

**Figure 1.**
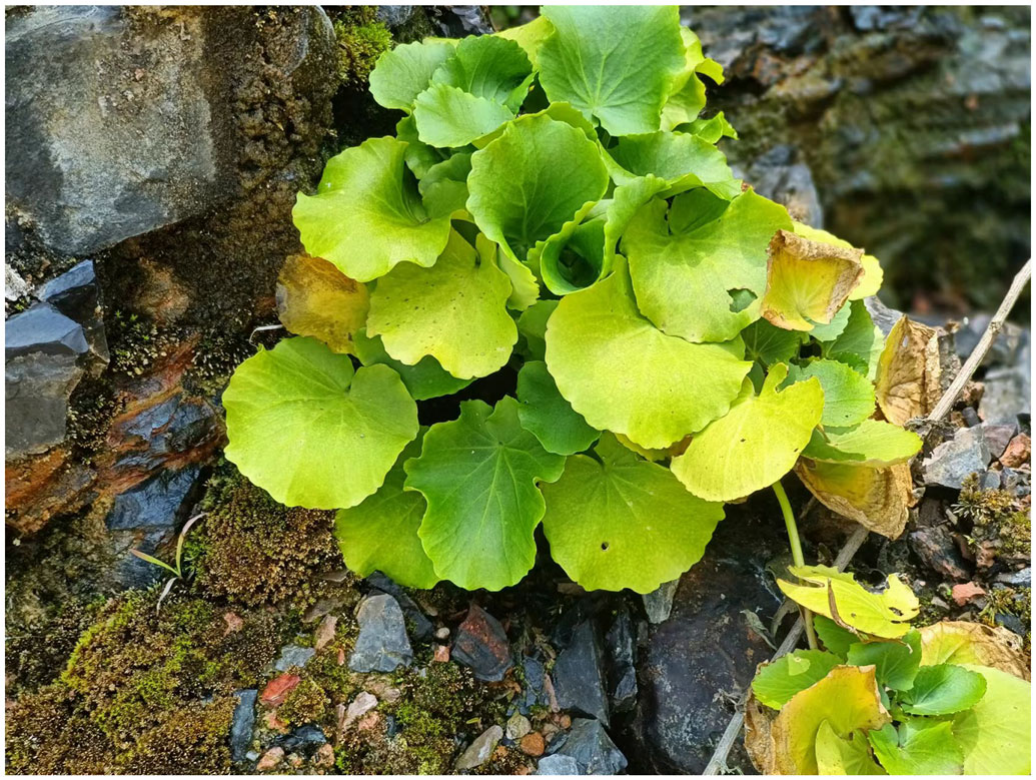
Morphological characteristics of wild conditions in *C. circaeoides*. Species photos were taken by the author in Wuyi Mountain, Enshi, Hubei, China, June 2020, without any copyright issues.

There are 154,838 base pairs in the cp genome of *C. circaeoides* (Figure 2). In the circular cp genome of *C. circaeoides*, a quadripartite structure was observed with an LSC (83,914 bp), an SSC (17,938 bp), and two IR regions (IRA and IRB, each of 26,493 bp). A total of 36.21% of *C. circaeoides’* cp genome is composed of GCs (LSC, 33.96%; SSC, 29.09%; IR, 42.35%). GC content and gene order were similar to those of *Cardamine amaraeform* (Raman et al. 2021). A total of 130 genes were found in the cp genome, including 85 protein-coding genes, 37 tRNA genes, and 8 rRNA genes. The introns were present in the following 12 protein-coding genes (*atp*F, *clp*P1, *ndh*A, *ndh*B, *paf*I, *pet*B, *pet*D, *rpl*16, *rpl*2, *rpo*C1, *rps*16), and 6 tRNA genes (*trn*A-UGC, *trn*G-UCC, *trn*I-GAU, *trn*K-UUU, *trn*L-UAA, and *trn*V-UAC). In *C. circaeoides*, 13 cis-clipping genes including *rps*16, *atp*F, *rpo*C1, *paf*I, *clp*P1, *pet*B, *pet*D, *rpl*16, *rpl*2(2), *ndh*B(2), *ndh*A, and a trans-splicing gene *rps12* were detected by CPGview-RSG (http://www.herbalgenomics.org/cpgview/). In addition, 95 SSRs were identified, including 89 single nucleotides (A/C/T, 93.68%) and 5 double nucleotides (AT/TA, 6.32%). At the same time, 23 tandem repeats were identified, with a total length of 15–42 bp. 33 dispersed repeats were identified with a threshold of 1E-4, and the length of repeat units ranged from 30 to 67.

**Figure 2.**
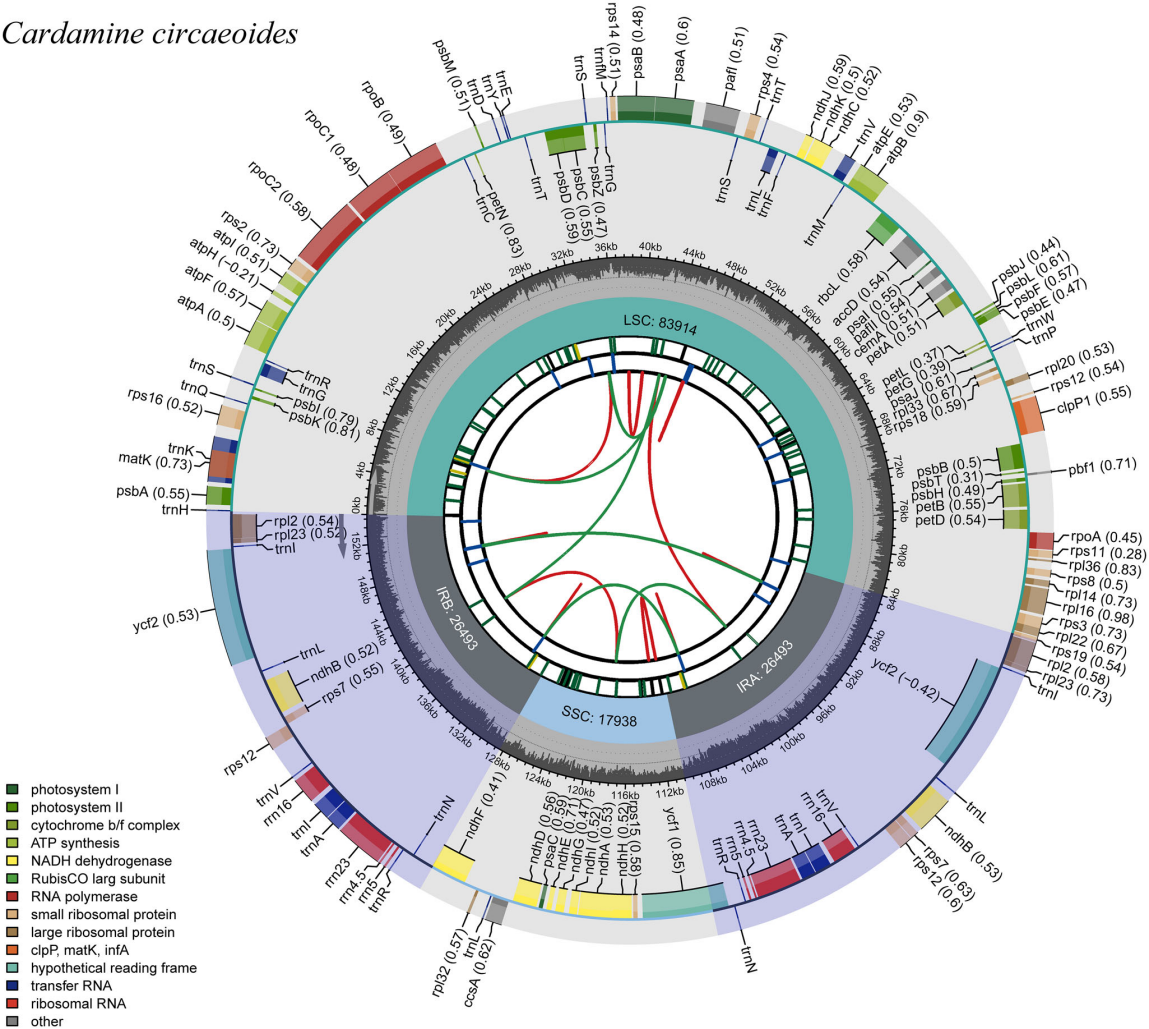
Graphic representation of features identified in *C. circaeoides* plastome by using CPGview-RSG (http://www.herbalgenomics.org/cpgview). The map contains seven circles. From the center going outward, the first circle shows the distributed repeats connected with red (the forward direction) and green (the reverse direction) arcs. The next circle shows the tandem repeats marked with short bars. The third circle shows the microsatellite sequences as short bars. The fourth circle shows the size of the LSC and SSC. The fifth circle shows the IRA and IRB. The sixth circle shows the GC contents along the plastome. The seventh circle shows the genes having different colors based on their functional groups.

For the purpose of analyzing the phylogenetic placement of *C. circaeoides* within *Cardamine*, we used the cp genome sequence of *C. circaeoides* and 31 other related Brassicaceae species, *Carica papaya*, and *Tarenaya hassleriana* interior as an outgroup to construct the phylogenetic tree. In order to align the sequences, MAFFT v7.450 was used with the default parameters (Katoh and Standley 2013). A maximum likelihood (ML) tree was then constructed using MEGE version X with a Generalized Time-Reversible (GTR) model, with 1000 bootstrap replications. According to the phylogenetic tree (Figure 3), the species of the Cardamine genera are monophyletic. Phylogenetic analysis demonstrated that *C. circaeoides* is closely related to *Cardamine hupingshanensis* with a strong bootstrap value (98.8% for ML).

**Figure 3.**
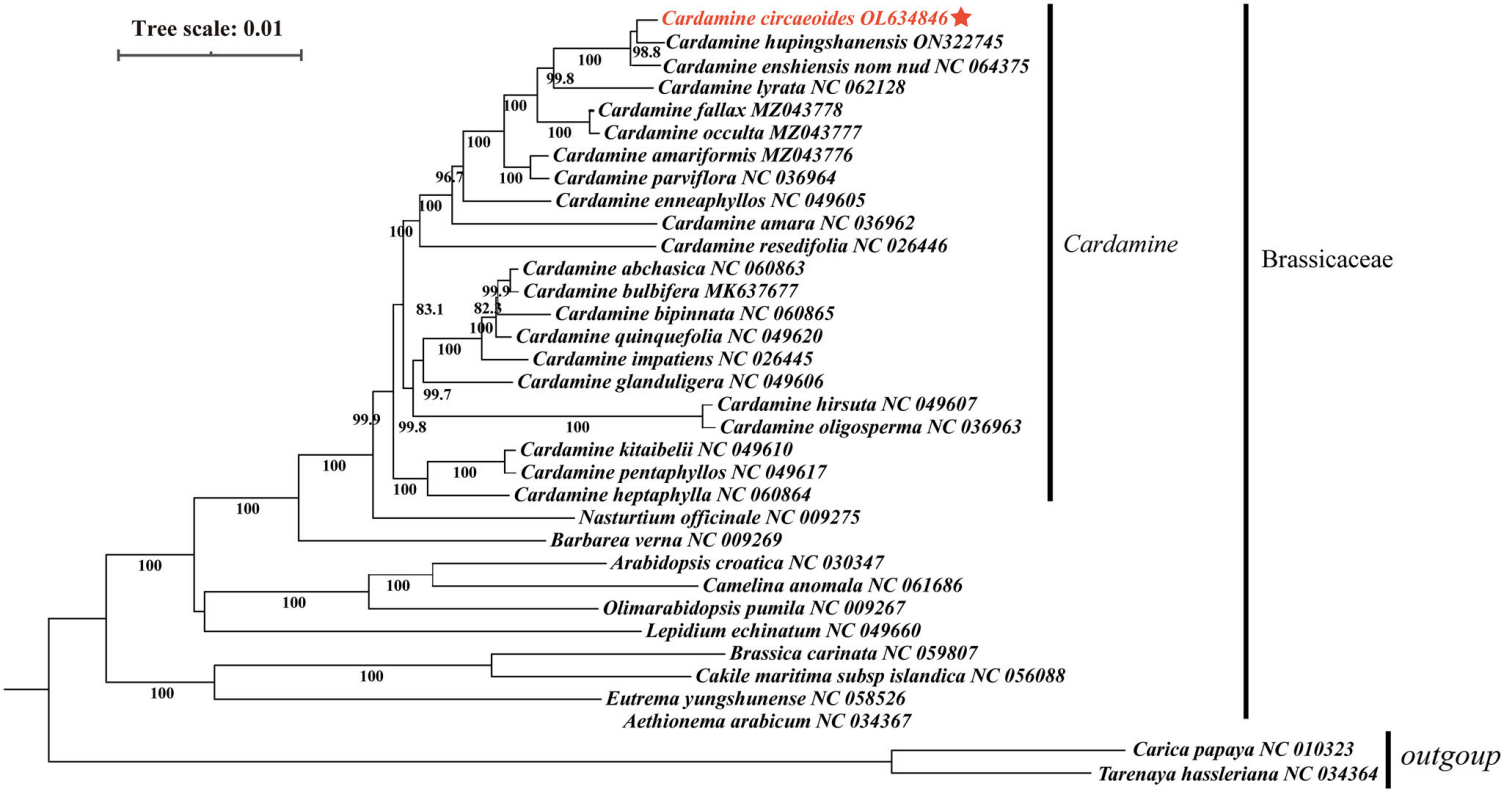
Based on 34 chloroplast genome sequences, a maximum-likelihood phylogenetic tree was constructed for *C. circaeoides*. A bootstrap value is indicated by the number on each node.

## Data Availability

These findings are supported by genome sequence data available in GenBank of NCBI (https://www.ncbi.nlm.nih.gov/) under accession number OL634846, associated BioProject, SRA, Bio-Sample numbers are PRJNA867123, SRR20954704, and SAMN30184410, respectively.
